# Influence of Organizational Learning and Dynamic Capability on Organizational Performance of Human Resource Service Enterprises: Moderation Effect of Technology Environment and Market Environment

**DOI:** 10.3389/fpsyg.2022.889327

**Published:** 2022-04-29

**Authors:** Shuilin Chen, Jianguo Zheng

**Affiliations:** Glorious Sun School of Business and Management, Donghua University, Shanghai, China

**Keywords:** organizational learning, dynamic capability, human resource service enterprises, organizational performance, technology environment and market environment

## Abstract

This study aims to explore the influence of organizational learning and dynamic capability on organizational performance of human resource service enterprises with the moderating role of technology environment and market environment. Data were gathered from 360 human resource service enterprises, and applied the hierarchical linear regression method and structural equation model to test the hypotheses. We found that organizational learning has a significantly positive impact on resource integration capability, as well as has a significantly positive impact on resource reconfiguration capability of human resource service enterprises. Resource integration capability and resource reconfiguration capability have a significantly positive impact on organizational performance. Moreover, results indicated that the resource integration capability and resource reconfiguration capability partially mediate in the relationship between organizational learning and organizational performance. Furthermore, technology environment and market environment have positive moderation effect between resource integration capability and organizational performance of human resource service enterprises, as well as have positive moderation effect between resource reconfiguration capability and organizational performance of human resource service enterprises. The current study contributes to a better understand the impact mechanism of organizational learning on organizational performance from the perspective of organizational learning theory and dynamic capability theory. In addition, this study provides implications for human resource service enterprises and managers to improve organizational performance.

## Introduction

In recent years, a new round of information technology revolution represented by big data, the internet and cloud computing has created new opportunities for the reform of the human resource service industry. Human resource service products, management models, and business models have been continuously innovated. The rapid differentiation and combination not only promotes the development of the industry from scale expansion to qualitative development, but also improves to the high-end service direction of the value chain. Organizational learning focuses on the exploration of future events and activities (Lampel et al., 2009), acquires new resources and changes in organizational capability for enterprises (Davies and Brady, 2000), helps enterprises to promote organizational performance from a strategic height, and is considered a long-term cultivation of continuous and breakthrough process of innovation (Levitt and March, 1988). In a highly uncertain market environment, organizational learning has important guiding significance for human resource service enterprises that are gradually implementing internationalization strategies in the context of economic globalization (Nurzaman et al., 2020). However, few researches deeply examine the management practice and internal mechanism of human resource service industry to achieve organizational performance through organizational learning. Organizational performance is closely related to its capability to acquire and utilize knowledge resources (Subramaniam and Youndt, 2005). Existing studies have recognized the critical role of knowledge in organizational performance (Zahra et al., 1999; Fleming, 2001; Zaied et al., 2012; Abubakar et al., 2019; Ode and Ayavoo, 2020). However, it fails to establish an effective link between knowledge activities and organizational performance, making the research on how enterprise knowledge activities affect organizational performance is still controversial (Gusmão et al., 2018; Oyemomi et al., 2019). Why the same organizational learning can lead to different performance results (Rehman et al., 2019; Singh et al., 2021)? How can enterprises improve organizational performance through organizational learning and realize the transformation from knowledge advantage to competitive advantage is a research theme that needs continuous attention.

Organizational learning, as the most popular perspective for examining organizational performance, has achieved many results, but it still fails to clearly reveal the knowledge base of organizational performance changes. The main reason is that scholars’ research on organizational learning focuses more on the acquisition of organizational knowledge and the continuous transformation of learning outcomes, which is mainly reflected in the dynamic process of discovering knowledge, using knowledge, and creating knowledge (Antunes and Pinheiro, 2020; Bilan et al., 2020). However, it fails to effectively reveal the conditions and mechanism of knowledge resources to create enterprise’s competitive advantage. Human resource service enterprises have some problems such as low resource integration and reconfiguration capability, relatively single integration methods, and low performance, which severely restrict the development of human resource service enterprises (Qian et al., 2019; Sun, 2019). At present, big data and artificial intelligence have impacted the recruitment, training, performance appraisal, and salary of human resource management, which comprehensively affected the development of the human resource service industry. For example, in the artificial intelligence recruitment competition held by North American headhunting enterprise SourceCon, the robot only took 3.2 s to screen out suitable resumes, which was 28,124 times faster than the top headhunting team. In the training field, big data, 3D virtual simulation technology and the extensive use of AR simulation learning scenarios helps to improve the learning effect of trainees (Xiao et al., 2018). Therefore, for human resource service enterprises, it is urgent to examine the internal relationship between their organizational learning logic and organizational performance from the strategic level.

Furthermore, organizational learning is a learning behavior and process that can bring and enhance the long-term adaptability of an enterprise (Levitt and March, 1988), enabling the enterprise to break through the current strategic path and enhance its core competitiveness (Real et al., 2006; Mueller et al., 2012; Mahdi et al., 2019; Dhir et al., 2020). It is considered to be the process by which enterprises cultivate long-term sustainable, breakthrough innovation, thereby improving organizational performance (Levitt and March, 1988). In fact, in the big data environment, the problems faced by human resource service enterprises are complex and changeable, and enterprises need to have the capability to continuously absorb, update and utilize new knowledge. As a higher-order capability, the essence of dynamic capability is the behavior orientation of improving, updating, reconstructing and recreating resources capability (Teece, 2007). Further, organizational learning is the key to changing and restructuring the operational capability of the human resource service industry (Winter, 2003; Ferreira et al., 2021; Pulsiri and Vatananan-Thesenvitz, 2021). Dynamic competencies and their frameworks provide a solid theoretical framework for integrating organizational learning theory in this study (Vera et al., 2011), so that this study can further explore the impact mechanism of organizational learning on organizational performance.

In view of the above study gaps and discussions, this study innovatively introduces a dynamic capability framework, applies organizational learning from the perspective of enterprise strategic integration as a process knowledge activity, and explorably proposes that organizational learning mediated by dynamic capability affects organizational performance hypothetical model, which will help to further reveal the black box of the relationship between organizational learning and enterprise organizational performance. At the same time, in order to clarify the role of environmental dynamics, this study attempts to use technology environment and market environment as moderator variables to analyze how to influence the relationship between dynamic capability and organizational performance.

The remaining parts of this study are organized as follows: Section “Theory and Hypotheses Development” focuses on hypotheses development, and proposes a hypothetical model that organizational learning and dynamic capability affect enterprise organizational performance. Section “Research Design” presents the study design, including data collection, and measurement methods. Section “Data Analysis” shows the empirical test results. Section “Discussion” discusses the findings of the study. Section “Conclusion” concludes this study, gives theoretical contributions and practical implications, while discusses limitations and future research directions.

## Theory and Hypotheses Development

### Effect of Organizational Learning on Dynamic Capability

Sáez et al. (2013) believed that knowledge theory was a combination of knowledge stock, and organizations need to continuously replenish new knowledge in order to improve performance. Organizational learning is a process in which an enterprise achieves the full use of resources to improve organizational behavior through the four links of acquiring knowledge, sharing knowledge, applying knowledge, and remembering knowledge, so as to maintain a sustainable competitive advantage (Ma et al., 2017). Dynamic capability theory points out that in order to maintain their leading position and market dominance, enterprises should formulate dynamic strategic goals, and based on this, cultivate dynamic capability that can effectively respond to changes in internal and external environments and resource restructuring, so as to take appropriate market behaviors. Therefore, in a dynamic competitive environment, having dynamic capability is critical to organizational performance (Wu, 2016). Dynamic capability can be divided into two dimensions: resource integration capability and resource reconfiguration capability (Teece, 2007). When an enterprise absorbs knowledge from the external environment, it not only increases the amount of the original resources of the enterprise, but also increases the types of enterprise resources. In the process of absorbing knowledge, enterprises increase the opportunities for information exchange with the outside world, obtain rich information, and then increase the ways for enterprises to obtain resources, so as to prepare for the process of enterprise resource integration. Enterprises acquire knowledge from within, mainly by accumulating their own management experience, organizational conventions, work processes, which is conducive to the formation of a fixed business model or path dependence effect, which leads to the tendency of enterprises to adopt the method of resource integration. In a word, organizational learning transforms organizational structure and power, making knowledge and information more accessible, faster, and more effective in the various activities of the organization. By encouraging the exchange, learning and sharing of knowledge between the organization and the environment, and promote enterprises resource integration capability.

The positive impact of organizational learning on the formation of dynamic capability of enterprises mainly includes the following two aspects: Firstly, by absorbing the knowledge of the internal and external environment of the organization, strengthening the degree of association between upstream and downstream enterprises, and integrating corporate resources. Secondly, by sharing and using the improvement of employees’ internal learning capability to improve the enterprise’s resource reconfiguration and rapid response capability (Persic et al., 2014). Numerous empirical studies have shown that organizational learning has a significant positive impact on the improvement of dynamic capability (Jiao et al., 2010; Villar et al., 2014; Wamba et al., 2017; Santoro et al., 2019; Ferreira et al., 2021). Yi et al. (2018) based on the empirical results of 213 Chinese enterprises, showed that ambidextrous learning has a positive impact on dynamic capability. Tu and Wu (2021) found that organizational learning can promote dynamic capability, and the combination of macro organizational learning is conducive to enhancing the competitive advantage of enterprises. Therefore, through organizational learning, innovating service products, grasping market opportunities, restructuring the value chain, reducing costs, optimizing resource allocation, and enhancing the core competitiveness of enterprises. Accordingly, we propose the following hypothesis:

**Hypothesis 1a**: Organizational learning has a positive impact on enterprise resource integration capability.

**Hypothesis 1b**: Organizational learning has a positive impact on enterprise resource reconfiguration capability.

### Effect of Dynamic Capability on Organizational Performance

Dynamic capability theory points out that the dynamic capability of enterprises can be improved through resource reconfiguration (Hamid Hawass, 2010). Dynamic capability are embedded in organizational processes, which can not only help adapt to changing environments, but also bring competitive advantages to the enterprise, thereby improving organizational performance (Lu and Guo, 2018; Cheng et al., 2019). For different enterprises, resource integration has different contents. Only by integrating their own resources and the reality of the market, can the resource allocation of enterprises be optimized. The unique resources owned by human resource service enterprises must be effectively reconstructed in order to form the core competitive advantage of the enterprise, gain long-term vitality in the market (Lin et al., 2005), and produce high organizational performance. Resource reconfiguration capability is to rationally allocate and recombine various resources owned by the enterprise in time and space to maximize the utility of resources. It is worth mentioning that the maximization of the utility of this kind of resources is not a simple allocation of resources, but a unique way of resource reconfiguration through creative reconfiguration planning, giving full play to the potential value of enterprise resources and realizing the competitive advantage of enterprises, thereby improving organizational performance.

This study argues that dynamic capability has a positive impact on organizational performance, which are the basis for improving organizational performance (Fainshmidt et al., 2016; Kareem and Alameer, 2019; Mikalef et al., 2020). Throughout the existing research, the successful construction of dynamic capability is very necessary to continuously improve the performance of enterprises (Wilden et al., 2013; Makkonen et al., 2014; Osisioma et al., 2016; Torres et al., 2018), because dynamic capability can follow the changes of the market, reconstruct the operational capability of organizations to adapt to changes through the integration and utilization of resources (Hill and Rothaermel, 2003). As a high-level capability of an organization, dynamic capability can change, develop and reconstruct enterprise resources, thereby improving organizational performance (Ambrosini and Bowman, 2009). Accordingly, we propose the following hypothesis:

**Hypothesis 2a**: Resource integration capability has a positive impact on enterprise organizational performance.

**Hypothesis 2b**: Resource reconfiguration capability has a positive impact on enterprise organizational performance.

### Mediation Effect of Dynamic Capability

When an enterprise conducts organizational learning, it not only fully taps the existing resource capability, but also uses the relationship network to obtain resources that it lacks, so that more and more resources are available to the enterprise (Stam and Elfring, 2008). At the same, these resources are increasingly dependent on a high level of scientific and technical knowledge (Song et al., 2019). In a complex and volatile hyper-competitive environment, enterprises need dynamic capability to sense and respond to market demands in a timely manner, integrate internal and external knowledge, other resources and capability in a timely manner, and improve organizational performance (Jiang et al., 2019; Qiu et al., 2020). Dynamic capability framework attempts to explain how an enterprise can flexibly respond to industrial changes through resource integration and resource reconfiguration capability without losing its competitive advantage. From this perspective, dynamic capability can integrate knowledge as a key resource for competitive advantage to improve organizational performance. Therefore, whether an enterprise’s organizational performance can be significantly improved not only depends on the enterprise’s capability to acquire and integrate new knowledge, but also depends on the enterprise’s capability to reconstruct external knowledge. This requires enterprises to have the dynamic capability to integrate internal and external knowledge and other resources, and to reconstruct internal and external resources, so that enterprises can improve organizational performance in management thinking and models.

Further, the improvement of enterprise organizational performance requires not only high-level organizational learning, but also strong dynamic capability. However, the impact of organizational learning on organizational performance needs to be achieved through the mediation effect of dynamic capability (Torres et al., 2018; Bogers et al., 2019; Ferreira et al., 2020; Li et al., 2020; Qader et al., 2022). Boccardelli and Magnusson (2006) explored the relationship between resource integration capability and organizational performance, and found that resource integration capability not only has a significant direct positive impact on organizational performance, but also has an indirect positive impact between organizational learning and organizational performance. Li et al. (2009) based on the sample data of 120 internet enterprises showed that the resource integration and reconfiguration capability of enterprise will affect its organizational performance. Hu et al. (2019) took fresh retail enterprises as an example and found that the quality of online and offline channel resource integration has a partial mediation effect between organizational learning capability and organizational performance. Aminu and Mahmood (2015) found that the implementation of dynamic capability in the competitive environment of manufacturing enterprises can effectively improve enterprises organizational performance. Therefore, this study believes that dynamic capability not only has a positive impact on the organizational performance of human resource service enterprises, but also may plays a mediation effect between organizational learning of human resource service enterprises and organizational performance. Accordingly, we propose the following hypothesis:

**Hypothesis 3a**: Resource integration capability has a mediation effect between organizational learning and organizational performance.

**Hypothesis 3b**: Resource reconfiguration capability has a mediation effect between organizational learning on and organizational performance.

### Moderation Effect of Environmental Dynamics

Environmental dynamics refers to the uncertainty caused by fluctuations in the external environment to the enterprise’s internal operating activities (Tian et al., 2018). The contingency theory points out that the production and operation activities of enterprises will be affected by multiple factors such as the environment, and enterprises should adjust their production and operation activities appropriately according to changes in the environment (Luthans and Stewart, 1977). Kohli and Jaworski (1990) scholars divided environmental dynamics into technology environment and market environment. Among them, technology environment can accelerate enterprise technology change, and technological evolution path is difficult to predict (Antoci et al., 2012). Market environment can accelerate changes in customer preferences, making it difficult for enterprises to accurately grasp customer satisfaction in a short period of time (Laskin, 2000). When the technology environment are high, the business environment of the enterprise changes rapidly, and the technology update speed is also faster, which increases the difficulty for enterprises to acquire knowledge. But it is relatively easier for enterprises to acquire new knowledge, so the resource integration capability has been significantly enhanced (Osisioma et al., 2016; Petrus, 2019). When the external knowledge is updated rapidly, affected by the technology environment, it is easier for enterprises to have a new understanding of the old knowledge that they have mastered. Therefore, the resource reconfiguration capability will also be strengthened (Romme et al., 2010; Rengkung, 2018). When the dynamics of the external technology environment is low, it is difficult for enterprises to feel the changes in the external environment, and the slow technological update speed makes it difficult for enterprises to acquire new knowledge, thus reducing resource integration capability (Cezarino et al., 2019; Knobbe and Proff, 2020). When resource integration capability is weakened, the enterprise will focus on the resource reconfiguration capability. However, it is difficult to absorb new ideas, the enterprise can no longer adapt to the current development of the enterprise using the past resource reconfiguration capability. Therefore, the enterprise resource reconfiguration capability is also will decrease (Battisti and Deakins, 2017; Wang and Hsu, 2018; Bitencourt et al., 2020).

In previous studies, it was found that dynamic capability and enterprises organizational performance is affected by the positive adjustment of environmental dynamics (Wu, 2010; Drnevich and Kriauciunas, 2011; Yue and Yu, 2019). With the rapid development of technology, market fluctuations, personalized customer needs, changes in macro policies, the threat of alternative products, the rapid improvement of competitors’ service quality and technological catch-up, the impact of the technology environment cannot be ignored, making human resource service enterprises need to pay full attention to technology environment (Lemos and Morehouse, 2005). Accordingly, we propose the following hypothesis:

**Hypothesis 4a**: Technology environment has a positive moderation effect between resource integration capability and organizational performance.

**Hypothesis 4b**: Technology environment has a positive moderation effect between resource reconfiguration capability and organizational performance.

Salvato and Vassolo (2018) believed that market environment increase and resource integration opportunities are fleeting, and enterprises would face more intense competition. Moreover, the resource integration capability is very important in acquiring customer demand and industry information, and its effect on enterprises organizational performance is also more significant. The higher market environment, the more efforts to obtain the knowledge and resources required by the enterprise through formal and informal networks, and integration of industrial chain resources are conducive to the improvement of organizational performance (Andersen et al., 2013). Therefore, market environment could enhance the impact of resource integration capability on organizational performance. For enterprise organizational change, rapidly changing market demands, government policies, industry structures and competitor strategies are the driving force for organizational change (Hai and Cao, 2014), and it is to adapt to the dynamic changes in the market environment that enterprises make organizational changes. The full range of market information and resource reconfiguration capability bring advantages to the process and direction of organizational change, enabling enterprises to recombine, restructuring and reallocating resources, thereby realizing the impact of resource reconfiguration capability on organizational performance. Accordingly, we propose the following hypothesis:

**Hypothesis 4c**: Market environment has a positive moderation effect between resource integration capability and organizational performance.

**Hypothesis 4d**: Market environment has a positive moderation effect between resource reconfiguration capability and organizational performance.

**FIGURE 1:**
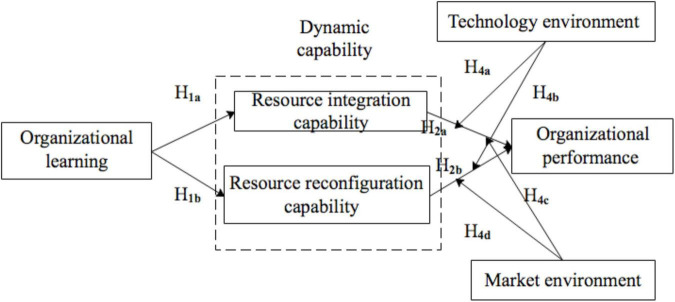
Research model.

Our theoretical research model is depicted in Figure 1.

## Research Design

### Participants and Procedure

Since data such as organizational learning, dynamic capability, and organizational performance cannot be obtained from public information, and this study data were collected through a convenience sampling technique. The items of the questionnaire are derived from academic papers published in high-level journals in the past, and are determined through discussions with experts in relevant research fields and middle-senior managers of enterprises. At the same time, the narration of the items is revised by means of English-Chinese translation to ensure that the questionnaire has good reliability and validity. Before the formal survey, this study first did a small sample data collection and pre-testing. The pre-testing stage can be divided into three steps: Firstly, after the initial survey questionnaire was determined, three middle-senior managers/technicians from different enterprises were invited to conduct in-depth structured interviews and revise the questions in the questionnaire based on their suggestions. Secondly, in order to ensure the readability of the questions in the questionnaire and the consistency of the item descriptions with the actual situation, a small-scale pre-testing was conducted among the enterprises in the park to ask their suggestions on the consistency of the item descriptions with the actual situation, and the data from these samples were analyzed to determine whether the scale had good reliability and validity. In addition, some expressions in the questionnaire were revised based on their suggestions. Thirdly, by revising the questionnaire guidelines and structural design of the questionnaire to form a formal questionnaire, and then conduct large-scale questionnaire distribution.

The target population in this study is the enterprises in China’s human resource service industrial park, and the questionnaires are filled out by middle-senior managers/technicians, mainly considering that the middle-senior managers/technicians have a clearer understanding of the technical situation of their own enterprises, and they are able to make more reasonable and comprehensive judgments on research questions. Previous literature also supports this approach, such as Ruppel and Harrington (2000) pointed out that the evaluation of organizational performance variables by middle-senior managers/technicians has certain theoretical and application basis.

There are two main forms of sending and receiving questionnaires. Firstly, fill in the answers on the spot, the researchers will distribute and recycle the paper version of the questionnaire on the spot. When multiple respondents fill in the questionnaires at the same time, the investigators will not make any annotations for sending and receiving the questionnaires, and will take them back in random order to fully protect the privacy of the respondents. Secondly, the electronic questionnaire is pushed to the sample enterprises in the form of WeChat, and the returned questionnaire does not need to be marked with the respondent’s name and other information. In order to further allow respondents to fully relax and answer truthfully, the purpose of study is explained in the first part of the questionnaire, it is promised that the collected data will only be used for academic research, and the relevant content will be kept confidential. The content that needs to be answered in the questionnaire mainly includes the background information of the enterprise (enterprise scale, enterprise nature, and enterprise service projects and so on), the identity of the respondents (confirm whether they belong to middle-senior managers/technicians), and test items for variables such as organizational learning, dynamic capability, and organizational performance.

In order to ensure the rigor and acceptable of the study, so the data collection period consisted of two stages: the first stage the sample data was collected from October 2018 to February 2019, and a total of 240 questionnaires were distributed; the second stage the sample data was collected from May to June 2020, and a total of 237 questionnaires were distributed. After separate ANOVA were performed on the sample data from the two stages, it was found that none of them were significantly different and therefore could be combined for analysis. This study collected a total of 477 questionnaires. Eliminate respondents who are not middle-senior managers/technicians, have serious missing information (such as questionnaires with three or more missing values), are not suitable for supplementary processing of missing values, untrue questionnaires, and answer regularly (such as more than five consecutive items). There are 360 valid questionnaires, and the effective rate of the questionnaire is 75.47%. The low recovery rate of the questionnaire is mainly due to the insufficient cooperation of some enterprise managers in the process of questionnaire collection. Of course, this is also a common phenomenon that exists when Chinese management researchers conduct questionnaire surveys (Tang and Li, 2016). Compared with the past literature, it is found that the effective rate of the questionnaire in this study is 75.47%, which is significantly higher than the sample recovery rate of 10 to 33% in empirical research (Vaccaro et al., 2012). This indicates that the questionnaire recovery rate of this study is within the acceptable range. In order to test the influence of sample selection bias, the questionnaire was divided into two parts (200 and 160) according to the time of answering, and an independent sample *t*-test was carried out. The results shows that there is no significant difference between the two parts of the questionnaire in terms of enterprise scale, enterprise nature, enterprise service items and so on, indicating that there is no obvious sample selection bias. The basic information of the sample are presented in Table 1.

**TABLE 1:** Basic characteristics of the sample.

Variable		Frequency	Percentage (%)
Enterprise size	20 people and below	94	26.11
	21–50 people	177	49.17
	51–100 people	53	14.72
	More than 100 people	36	10.00
Enterprise nature	State-owned enterprise	12	3.33
	Non-state-owned enterprise	348	96.67
Enterprise service items	Human resources software system	174	48.33
	Online recruitment	248	68.89
	Human resources outsourcing	328	91.11
	Mid-high end talent search	144	40.00
	Flexible employment	169	46.94
	Human resources comprehensive consultation	212	58.89
R&D department	Not exist	176	48.89
	Exist	184	51.11

Table 1 shows the results of basic enterprises characteristics. In terms of enterprise scale, enterprises with 21–50 employees are the majority, accounting for 49.17%. In terms of enterprise nature, 96.67% of the enterprise’s nature is attributable to non-state-owned enterprises. From the perspective of enterprise service projects, human resource service outsourcing and online recruitment are the mainstream service projects of human resource service enterprises, while service projects such as mid-high end talent search and flexible employment are relatively weak. 51.11% have a technology R&D department, while 48.89% do not have a technology R&D department. The results are presented in Table 1.

Although the data of this study were collected from two different stages, the data were collected through questionnaires, and the relevant data were provided by the middle-senior managers/technicians of each enterprise, and it was still necessary to carry out the common method bias test. In order to ensure that the test results are not seriously affected, and considering that the Harman’s singe factor test is an insensitive test method (Tang and Wen, 2020). Drawing on the practice of previous studies (Wei and Wu, 2013; Li et al., 2014), this study tested for common method bias by controlling for unmeasured single-method latent variables (Mathieu and Farr, 1991). This approach is to incorporate a single method into a model as a latent variable uncorrelated with other factors, allowing all observed variables to have loads on this latent variable. The results shows that the fitting index of the model after including the common method bias latent variable is better (χ^2^/*df* = 2.47, RMSEA = 0.06, CFI = 0.98, TLI = 0.96, and SRMR = 0.03). After inspection, the corrected RMSEA difference is less than the critical value of the 0.05 level, indicating that the fitting degree of the model without the common method bias latent variable is not significantly different from that with the common method bias latent variable. It means the common method bias of the study was not severe.

### Measures

The questionnaire scale of this study is mainly composed of four parts: organizational learning scale, dynamic capability scale, organizational performance scale, and environmental dynamics scale. The scales used in this study are all derived from academic papers published in high-level journals in the past, in order to ensure the quality of the scales. According to the characteristics of human resource service enterprises, make appropriate adjustments to the scale to make its expression more in line with the actual situation and understanding of the subjects. For the English scales involved in the study, according to the suggestion of Brislin (1980), this study adopts the translation and back-translation method to localize the scales. Scholars in related fields are invited to check the translation results to ensure the semantic equivalence of the Chinese scale and the original scale. Through the above process, the content of the questionnaire should be avoided to be instructive and vague, and efforts should be made to ensure that the research questionnaire conforms to academic norms and is easy for management practitioners to understand. On this basis, the final draft of the questionnaire is formed.

The organizational learning scale refers to the three items of the organizational learning measurement scale by Fu and Fu (2007) and Cai and Yin (2009). The dynamic capability scale mainly refers to Teece et al. (1997) and Zheng et al. (2010) measurement scale for dynamic capability, it contains six items in two dimensions: resource integration capability and resource reconfiguration capability. The enterprise organization performance scale refers to the three items of the research results of Fang (2008) and Souder and Jensen (2010). The environmental dynamics scale refers to the research on environmental dynamics by Jansen et al. (2006) and Wang (2003), it is divided into technology environment and market environment, combined with the external environment of the human resource service enterprises, a total of nine items are compiled. In addition, according to previous literature (Zhu and Yang, 2019; Zhu et al., 2019), enterprise size, enterprise nature, the service items, and whether there is a technology R&D department may all have an impact on enterprises organizational performance, this study uses these variables as control variables. At the same time, considering that the sample enterprises are distributed in six major service projects, including human resource software systems, online recruitment, human resource outsourcing, mid-high end talent search and flexible employment. This study sets up dummy variables to control the impact of service items on the organizational performance of human resource service enterprises. The above scales are all measured by Likert’s seven-level scale, the options were “completely disagree”(=1), “completely agree”(=7). The results are presented in Table 2.

**TABLE 2:** Related variables measurement items.

Variable		Item		Main source
Organizational learning		OS1	Human resource service enterprises employees have clear mission goals	Fu and Fu, 2007; Cai and Yin, 2009
		OS2	Managers make reasonable commitments for employees’ work behaviors	
		OS3	Serving enterprises to establish an internal learning sharing mechanism for employees	
Dynamic capability	Resource integration capability	RI1	Apply big data to collaborate well within the enterprise	Teece et al., 1997; Zheng et al., 2010
		RI2	Enhancing the correlation between upstream and downstream enterprises through big data	
		RI3	Establish a network of relationships with external parties to obtain resources	
	Resource reconfiguration capability	RR1	Human resource service enterprises have rapid response capability	
		RR2	Human resource service enterprises efficiently respond to policy changes	
		RR3	Organizational structure allows to break the rules to ensure flexibility	
Organizational performance		EP1	Enterprise sales revenue grows faster than peers	Fang, 2008; Souder and Jensen, 2010
		EP2	Corporate profitability is growing faster than peers	
		EP3	Enterprise market share is growing faster than peers	
Environmental dynamics	Technology environment	TD1	Technology R&D investment-high output efficiency	Wang, 2003; Jansen et al., 2006
		TD2	Have many patents	
		TD3	Implement human resources ISO9001:2000	
		TD4	Research funding accounts for a significant proportion of income	
		TD5	New technology use and achievement transformation	
	Market environment	MD1	Clear market positioning and product price attractiveness	
		MD2	High market share	
		MD3	Diversification of service products	
		MD4	Revenue accounts for a large share of the same industry	

## Data Analysis

### Reliability and Validity Test

Before the formal analysis of the sample data, the reliability and validity of the four scales of organizational learning, dynamic capability, organizational performance, and environmental dynamics were tested. The results are presented in Table 3.

**TABLE 3:** Reliability and validity analysis of variables.

Variable			Validity		Reliability	
			Load factor	AVE	α value	Combination reliability
Organizational learning		OS1	0.85	0.60	0.85	0.82
		OS2	0.74			
		OS3	0.73			
Dynamic capability	Resource integration capability	RI1	0.79	0.65	0.83	0.85
		RI2	0.84			
		RI3	0.78			
	Resource reconfiguration capability	RR1	0.85	0.68	0.87	0.87
		RR2	0.81			
		RR3	0.82			
Organizational performance		EP1	0.88	0.71	0.88	0.88
		EP2	0.86			
		EP3	0.79			
Environmental dynamics	Technology environment	TD1	0.76	0.63	0.90	0.89
		TD2	0.77			
		TD3	0.74			
		TD4	0.85			
		TD5	0.85			
	Market environment	MD1	0.77	0.66	0.88	0.88
		MD2	0.84			
		MD3	0.83			
		MD4	0.80			

From the results in Table 3, Cronbach’s α coefficient value of each variable is between 0.83 and 0.90 and exceeds 0.70, indicating that the sample data of the questionnaire has good reliability (Lord, 1955). The combined reliability value of each variable is between 0.82 and 0.89, indicating that the item has a strong explanatory power for each dimension. From the average variance extraction value, they are all between 0.60 and 0.71, which all are much greater than 0.5, indicating that the validity of the scale is good (Shepard, 1993). The results are presented in Table 3.

### Descriptive Statistics and Correlation Test of Variables

Descriptive statistics are performed on the six core variables involved in the study, and the correlation coefficient, mean and standard deviation results are presented in Table 4. The variance expansion factor of each variable is between 1 and 2, which far less than 10, indicating that multicollinearity is not serious. The results are presented in Table 4.

**TABLE 4:** Descriptive statistics and correlation test of variables.

Variable	1	2	3	4	5	6	7	8	9	10	11	12	13	14	15
1	1														
2	–0.01	1													
3	0.16**	−0.12*	1												
4	0.29**	0.06	–0.06	1											
5	0.11*	0.06	−0.19**	0.13*	1										
6	0.27**	0.16**	0.16**	0.15**	0.12*	1									
7	0.24**	0.14*	0.03	0.28**	0.27**	0.32**	1								
8	0.20**	0.06*	0.29**	0.09	–0.02	0.31**	0.29**	1							
9	0.22**	0.12*	0.18**	0.03	−0.11*	0.05	0.02	0.12*	1						
10	0.03	–0.02	0.16**	−0.12*	0.06	0.07	0.16*	0.06	0.01	1					
11	0.10	–0.01	0.22**	–0.02	0.01	0.11*	0.10	0.19**	0.08	0.64**	1				
12	0.02	–0.01	0.26**	−0.12*	0.03	0.08	0.14**	0.13*	0.07	0.73**	0.68**	1			
13	0.12*	–0.02	0.10	–0.06	0.13*	0.12*	0.13*	0.02	0.03	0.58**	0.51**	0.56**	1		
14	0.19**	−0.12*	0.27**	–0.07	–0.03	0.10	0.01	0.27**	0.11*	0.43**	0.53**	0.47**	0.47**	1	
15	0.12*	0.08	0.18**	−0.13*	–0.02	0.11*	0.08	0.15**	0.09	0.57**	0.54**	0.57**	0.56**	0.64**	1
Mean	2.09	0.03	0.48	0.69	0.91	0.40	0.47	0.59	0.51	5.27	5.21	5.03	5.13	5.00	5.12
SD	0.90	0.18	0.50	0.46	0.29	0.49	0.50	0.49	0.50	0.96	0.96	1.03	0.96	1.01	1.02

*p < 0.05 and **p < 0.01. SD is standard deviation, 1 is enterprise scale, 2 is enterprise nature, 3 is the human resource software system, 4 is the online recruitment, 5 is the human resource outsourcing, 6 is the mid-high end talent search, 7 is the flexible employment, 8 is the human resources comprehensive consultation, 9 is the R&D department, 10 is organizational learning, 11 is resource integration capability, 12 is resource reconfiguration capability, 13 is organizational performance, 14 is technology environment, and 15 is market environment.

The empirical results show that there is a significantly positive correlation between organizational learning and resource integration capability (*r* = 0.64, *p* < 0.01), hypothesis 1a has been initially verified. Organizational learning and resource reconfiguration capability show a significantly positive correlation (*r* = 0.73, *p* < 0.01), hypothesis 1b has been initially verified. Resource integration capability and organizational performance are significantly positive correlated (*r* = 0.51, *p* < 0.01), hypothesis 2a has been initially verified. There is a significantly positive correlation between resource reconfiguration capability and organizational performance (*r* = 0.56, *p* < 0.01), hypothesis 2b has been initially verified. In summary, these research results are consistent with the research hypothesis and provide preliminary evidence support for further research.

### Testing of Hypotheses

This study separately explored the impact of organizational learning on the two dimensions of dynamic capability (resource integration capability and resource reconfiguration capability). The regression analysis results are presented in Tables 5, 6. Table 5 performs hierarchical linear regression analysis with control variables, organizational learning and resource integration capability. The results of Model 1-2 show that after controlling for other variables, organizational learning has a significantly positive impact on resource integration capability (β = 0.645, *p* < 0.001), indicating that the hypothesis 1a has been verified. The results are presented in Table 5.

**TABLE 5:** Regression analysis results of organizational learning on resource integration capability.

Research variables		Dependent variable: Resource integration capability	
		Model 1-1	Model 1-2
Enterprise size		0.039	0.034
Enterprise nature		0.073	0.140
Enterprise service items	Human resources software system	0.329**	0.128
	Online recruitment	–0.116	0.110
	Human resources outsourcing	0.085	–0.032
	Mid-high end talent search	0.048	0.034
	Flexible employment	0.108	–0.140
	Human resources comprehensive consultation	0.222	0.262**
R&D department		0.044	0.058
Organizational learning			0.645***
*R* ^2^		0.074	0.453
Adjustment *R*^2^		0.050	0.437
Δ*R*^2^			0.379***
*F*		3.107**	28.890***

**p < 0.01 and ***p < 0.001.

**TABLE 6:** Regression analysis results of organizational learning on resource reconfiguration capability.

Research variables		Dependent variable: Resource reconfiguration capability	
		Model 1-1	Model 1-2
Enterprise size		–0.046	–0.051
Enterprise nature		0.008	0.085
Enterprise service items	Human resources software system	0.509***	0.274**
	Online recruitment	−0.321**	–0.057
	Human resources outsourcing	0.219	0.082
	Mid-high end talent search	0.010	–0.007
	Flexible employment	0.318**	0.029
	Human resources comprehensive consultation	0.062	0.109
R&D department			0.089
Organizational learning			0.752***
*R* ^2^		0.110	0.565
Adjustment *R*^2^		0.087	0.552
Δ*R*^2^			0.455***
*F*		4.812***	45.323***

**p < 0.01 and ***p < 0.001.

Table 6 performs hierarchical linear regression analysis with control variables, organizational learning and resource reconfiguration capability. The results of Model 2-2 show that after controlling for other variables, organizational learning has a significantly positive impact on resource reconfiguration capability (β = 0.752, *p* < 0.001), indicating that the hypothesis 1b has been verified. The results are presented in Table 6.

In order to test the impact of the two dimensions of dynamic capability (resource integration capability and resource reconfiguration capability) on the organization performance of human resource service enterprises, Table 7 uses the control variables, resource integration capability, and resource reconfiguration capability to perform a hierarchical linear regression analysis on the organization performance of the enterprise. From the results of Model 3-1, it can be found that resource integration capability has a significantly positive impact on organizational performance (β = 0.510, *p* < 0.001), indicating that the hypothesis 2a has been verified. From the results of Model 3-2, it can be found that resource reconfiguration capability has a significantly positive impact on organizational performance (β = 0.525, *p* < 0.001), indicating that the hypothesis 2b has been verified. Model 3-3 shows that the explanatory power of the model has been significantly improved (Δ*R*^2^ = 0.314, *p* < 0.001) after adding resource integration capability and resource reconfiguration capability at the same time on the basis of control variables. It shows that resource integration capability and resource reconfiguration capability have a significantly positive impact on the organizational performance of human resource service enterprises (β = 0.264, *p* < 0.001; β = 0.358, *p* < 0.001), which further shows that the hypothesis 2a and 2b are verified. The results are presented in Table 7.

**TABLE 7:** Regression analysis results of dynamic capability on organizational performance.

Research variables		Dependent variable: Organizational performance			
		Model 3-0	Model 3-1	Model 3-2	Model 3-3
Enterprise size		0.112	0.092	0.136*	0.118*
Enterprise nature		–0.124	–0.162	–0.128	–0.146
Enterprise service items	Human resources software system	0.192	0.024	–0.075	–0.077
	Online recruitment	−0.271*	−0.212*	–0.103	–0.126
	Human resources outsourcing	0.408*	0.364*	0.293	0.307*
	Mid-high end talent search	0.113	0.088	0.108	0.097
	Flexible employment	0.200	0.145	0.033	0.058
	Human resources comprehensive consultation	–0.122	−0.235*	–0.154	−0.202*
R&D department		0.015	–0.007	–0.023	–0.022
Resource integration capability			0.510***		0.264***
Resource reconfiguration capability				0.525***	0.358***
*R* ^2^		0.065	0.306	0.343	0.379
Adjustment *R*^2^		0.041	0.286	0.324	0.359
Δ*R*^2^			0.242***	0.278***	0.314***
*F*		2.693**	15.413***	18.186***	19.305***

*p < 0.05, **p < 0.01, and ***p < 0.001.

### Structural Equation Model Path Test

**FIGURE 2:**
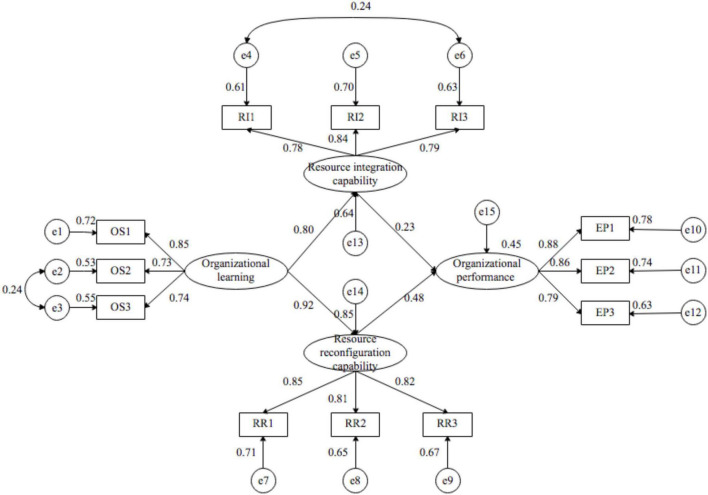
Model path diagram of organizational learning, dynamic capability, and organizational performance.

This study used AMOS22.0 to construct a structural equation model to analyze the internal mechanism between organizational learning, dynamic capability and human resource service enterprises organizational performance. The structural equation model is shown in Figure 2. The coefficients in the figure are all standardized coefficients. We conducted a model fit degree analysis (see Table 8). By referring to the fitting index standard defined by Wu and Ding (2004), the empirical results show that all measures have acceptable validity, the goodness of fit index (GFI) = 0.936 and the root mean square error approximation (RMSEA) = 0.078, the GFI statistics exceed the recommended threshold level of 0.90 and the RMSEA statistics within the recommended threshold level of 0.08. Therefore, the model-fitting level constructed in this study is acceptable. The results are presented in Table 8.

**TABLE 8:** Model fitting index values.

Statistical tests	Fitting indicators	Evaluation criterion	Model results	
Absolute fitness index	GFI	>0.90	0.936	Ideal
	AGFI	>0.90	0.895	Good
	RMSEA	<0.08	0.078	Ideal
Value-added fitness index	NFI	>0.90	0.946	Ideal
	IFI	>0.90	0.963	Ideal
	CFI	>0.90	0.962	Ideal
Minimalist fitness index	PGFI	>0.50	0.576	Ideal
	PNFI	>0.50	0.688	Ideal
	PCFI	>0.50	0.700	Ideal

*“Ideal” means that the fitting index is within the reference value range; “Good” means that the fitting index is not within the reference value range but is slightly lower or slightly higher.*

Table 9 show that organizational learning has a significantly positive impact on the resource integration capability of human resource service enterprises, and its path coefficient reached 0.802 (*p* < 0.001). Organizational learning has a significantly positive impact on the resource reconfiguration capability of human resource service enterprises, and its path coefficient reached 0.924 (*p* < 0.001). Resource integration capability has a significantly positive impact on organizational performance of human resource service enterprises, and its path coefficient reached 0.234 (*p* < 0.01). Resource reconfiguration capability has a significantly positive impact on organizational performance of human resource service enterprises, and its path coefficient reached 0.481 (*p* < 0.001). In summary, the research hypotheses 1a, 1b, 2a, and 2b have been further verified. The results are presented in Table 9 and Figure 2.

**TABLE 9:** Hypothesis test results of path coefficients of structural equation model.

Variable	Standardized path coefficients	*P*	Hypothesis	Result
Organizational learning → Resource integration capability	0.802	0.000	H**_1a_**	Supported
Organizational learning → Resource reconfiguration capability	0.924	0.000	H**_1b_**	Supported
Resource integration capability → Organizational performance	0.234	0.005	H**_2a_**	Supported
Resource reconfiguration capability → Organizational performance	0.481	0.000	H**_2b_**	Supported

In addition, it can be seen from Figure 2 that there may be a mediation effect between organizational learning and human resource service enterprises organizational performance. Therefore, the possible mediation effect in the above models are tested. In order to further test whether the resource integration capability has a mediation effect between organizational learning and organizational performance, and resource reconfiguration capability between organizational learning and organizational performance, this study did not use the traditional Sobel test method, mainly because the Sobel test has certain limitations (Mackinnon et al., 2007; Hayes, 2009). The premise hypothesis for the derivation of Sobel test statistics is: not only a and b conform to the normality hypothesis, but also *a* × *b* must conform to the normality hypothesis. Normally, the latter is difficult to test, so the accuracy of Sobel’s test results is often questioned. Therefore, this study adopts the Bootstrap confidence interval method with deviation correction to test, and the confidence level is set to 95% (Zhang and Kang, 2016).

From Table 10, the following conclusions can be drawn. Firstly, the results indicate that the indirect effect of organizational learning on human resource service enterprises organizational performance is (0.547, 0.734) at the 95% confidence level, zero is not within the range, and the *p*-value is less than 0.05, which indicates that the mediation effect of resource integration capability between organizational learning and organizational performance is significant. Moreover, the direct effect of organizational learning on organizational performance is (0.273, 0.527), zero is not within the range, and the *p*-value is less than 0.05, which further indicates that resource integration capability plays a partial mediation effect between organizational learning and organizational performance. Thus, Hypothesis 3a is supported. Secondly, the indirect effect of resource reconfiguration capability between organizational learning and organizational performance is (0.665, 0.830) at the 95% confidence level, zero is not within the range, and the *p*-value is less than 0.05, which indicates that the mediation effect of resource reconfiguration capability between organizational learning and organizational performance is significant. In addition, the direct effect of resource reconfiguration between organizational learning and organizational performance is (0.211, 0.493), zero is not within the range, and the *p*-value is less than 0.05, which further indicates that resource reconfiguration capability, it plays a partial mediation effect between organizational learning and organizational performance. Thus, Hypothesis 3b is supported. The results are presented in Table 10.

**TABLE 10:** Bootstrap mediation effects testing.

Summary of the hypothesized path	Coefficient	Mediation effect		Result
		95% deviation modified confidence interval		
		LL	UL	
Organizational learning → Organizational performance	0.566***	0.467	0.673	Significant
Organizational learning → Resource integration capability → Organizational performance	0.166***	0.547	0.734	Significant
Organizational learning → Resource reconfiguration capability → Organizational performance	0.216***	0.665	0.830	Significant
Organizational learning → Resource integration capability → Organizational performance	0.400***	0.273	0.527	Significant
Organizational learning → Resource reconfiguration capability → Organizational performance	0.350***	0.211	0.493	Significant

LL, lower limit; UL, upper limit. ***p < 0.001.

### Moderation Effect Test

In order to test whether environmental dynamics has a moderation effect between dynamic capability and human resource service enterprises organizational performance. In this study, the two dimensions of dynamic capability (resource integration capability and resource reconfiguration capability) and the two dimensions of moderation variable environmental dynamics (technology environment and market environment) are centrally processed and their interaction terms are calculated, then perform hierarchical linear regression, the results are presented in Table 11. Model 4-1 takes control variables, resource integration capability, and technology environment as independent variables, and uses organizational performance as dependent variables for regression analysis. From the results of Model 4-1, it can be seen that resource integration capability has a significant positive impact on organizational performance (β = 0.365, *p* < 0.001), and technology environment also has a significant impact on organizational performance (β = 0.293, *p* < 0.001), which shows that resource integration capability and technology environment are significant to the organizational performance of human resource service enterprises. Model 4-2 adds resource integration capability and technology environment interaction terms on the basis of Model 4-1, the explanatory power of the model has been significantly improved (Δ*R*^2^ = 0.020, *p* < 0.001), explain that the model’s interpretation of organizational performance has increased, and technology environment has positively moderation effect between resource integration capability and organizational performance (β = 0.104, *p* < 0.01). Therefore, the Hypothesis 4a has been verified. Model 4-3 takes control variables, resource reconfiguration capability and technology environment as independent variables, and uses organizational performance as dependent variables for regression analysis. It can be seen from the results of Model 4-3 that resource reconfiguration capability has a significant positive impact on organizational performance (β = 0.403, *p* < 0.001), and technology environment also has a significant impact on organizational performance (β = 0.289, *p* < 0.001), which shows that resource reconfiguration capability and technology environment are both significant to the organizational performance of human resource service enterprises. Model 4-4 adds the interactive terms of resource reconfiguration capability and technology environment on the basis of model 4-3, the explanatory power of the model has been significantly improved (Δ*R*^2^ = 0.018, *p* < 0.001), it shows that the interpretation level of the model for organizational performance has increased, and the technology environment has positively moderation effect between resource reconfiguration capability and organizational performance (β = 0.090, *p* < 0.01). Therefore, the Hypothesis 4b has been verified. The results are presented in Table 11.

**TABLE 11:** The moderation effect of dynamic capability on organizational performance.

Research variables		Dependent variable: Organizational performance							
		Model 4-1	Model 4-2	Model 4-3	Model 4-4	Model 4-5	Model 4-6	Model 4-7	Model 4-8
Enterprise size		0.047	0.033	0.081	0.074	0.049	0.046	0.080	0.088
Enterprise nature		0.027	–0.090	0.048	–0.014	–0.346	–0.357	–0.310	–0.292
Enterprise service items	Human resources software system	–0.028	–0.006	–0.111	–0.084	–0.011	–0.004	–0.072	–0.064
	Online recruitment	–0.161	–0.164	–0.076	–0.080	–0.074	–0.105	–0.016	–0.054
	Human resources outsourcing	0.340*	0.325*	0.283	0.272	0.397**	0.358*	0.348*	0.336*
	Mid-high end talent search	0.086	0.096	0.100	0.105	0.079	0.089	0.093	0.089
	Flexible employment	0.203*	0.187*	0.114	0.091	0.115	0.115	0.046	0.037
	Human resources comprehensive consultation	−0.331**	−0.333***	−0.273**	−0.249**	−0.242**	−0.267**	−0.191*	−0.178*
R&D department		–0.022	–0.016	–0.035	–0.028	–0.012	–0.003	–0.022	0.010
Resource integration capability		0.365***	0.368***			0.304***	0.318***		
Resource reconfiguration capability				0.403***	0.414***			0.333***	0.364***
Technology environment		0.293***	0.303***	0.289***	0.273***				
Resource integration capability ×Technology environment			0.104**						
Resource reconfiguration capability ×Technology environment					0.090**				
Market environment						0.382***	0.375***	0.352***	0.321***
Resource integration capability ×Market environment							0.104**		
Resource reconfiguration capability ×Market environment									0.096***
*R*_2_		0.366	0.386	0.404	0.423	0.415	0.433	0.430	0.451
Adjustment *R*^2^		0.346	0.365	0.385	0.403	0.396	0.413	0.412	0.432
Δ*R*^2^			0.020		0.018		0.018		0.020
*F*		18.260***	18.167***	21.471***	21.161***	22.414***	22.048***	23.890***	23.714***

*p < 0.05, **p < 0.01, and ***p < 0.001.

Model 4-5 takes control variables, resource integration capability and market environment as independent variables, and uses organizational performance as dependent variables for regression analysis. From the results of Model 4-5, it can be seen that resource integration capability has a significant positive impact on organizational performance (β = 0.304, *p* < 0.001), and market environment also has a significant impact on organizational performance (β = 0.382, *p* < 0.001), which shows that resource integration capability and market environment are both significant to organizational performance. Model 4-6 adds resource integration capability and market environment interaction terms on the basis of Model 4-5, the explanatory power of the model has been significantly improved (Δ*R*^2^ = 0.018, *p* < 0.001), explain that the model’s interpretation of organizational performance has increased, and market environment has positively moderation effect between resource integration capability and organizational performance (β = 0.104, *p* < 0.01). Therefore, the Hypothesis 4c has been verified. Models 4-7 take control variables, resource reconfiguration capability and market environment as independent variables, and use organizational performance as dependent variables for regression analysis. From the results of models 4-7, it can be seen that resource reconfiguration capability has a significant positive impact on organizational performance (β = 0.333, *p* < 0.001), and market environment also has a significant impact on organizational performance (β = 0.352, *p* < 0.001), which shows that resource reconfiguration capability and market environment are both significant to the organizational performance of human resource service enterprises. Model 4-8 adds the interaction terms of resource reconfiguration capability and market environment on the basis of Model 4-7, the explanatory power of the model has been significantly improved (Δ*R*^2^ = 0.020, *p* < 0.001), explain that the model’s interpretation of organizational performance has increased, and market environment has positively moderation effect between resource reconfiguration capability and organizational performance (β = 0.096, *p* < 0.001). Therefore, the Hypothesis 4d was supported. The results are presented in Table 11.

## Discussion

With the evolution of organizational knowledge as the core, this study explores the impact of organizational learning on organizational performance of human resource service enterprises under the unified framework of dynamic capability. Exploringly proposes an integrated model of the relationship between organizational learning and organizational performance with dynamic capability as the mediation variable and environmental dynamics as the moderation variable. On the basis of this model, the hypothesis of the correlation between the above constructs is put forward, and the hypothesis is tested through the empirical analysis of 360 valid questionnaire data. The empirical findings are discussed below.

Firstly, when exploring the impact of organizational learning on the organizational performance of human resource service enterprises, this study finds that organizational learning has a significant positive impact on the dynamic capability of human resource service enterprises and its two dimensions, which is basically consistent with the conclusions of existing research (Giniuniene and Jurksiene, 2015; Bhatia, 2021; Matarazzo et al., 2021). The more valuable finding of this study is that organizational learning has a smaller impact on resource integration capability than resource reconfiguration capability. This conclusion shows that dynamic capability have different positions and roles in the relationship between organizational learning and organizational performance of human resource service enterprises. In the process of organizational learning affecting organizational performance, organizational learning can overcome the knowledge acquisition dilemma caused by knowledge characteristics and organizational boundaries, and provide the preparation of heterogeneous knowledge resources for the improvement of organizational performance. To promote the upgrading or transformation of technology and management of enterprises, so that enterprises can take the lead in expanding existing markets or developing new markets in the competition, which is conducive to the improvement of enterprise performance.

Secondly, organizational learning has a significantly positive impact on the dynamic capability of human resource service enterprises and its various dimensions. Moreover, dynamic capability and its dimensions have a significantly positive impact on the organizational performance of human resource service enterprises, which is basically consistent with the conclusions of existing research (Fainshmidt et al., 2016; Takahashi et al., 2017; Zhou et al., 2019; Sun et al., 2021). However, the more meaningful finding of this study is that dynamic capability has a significantly partial mediation effect between organizational learning and organizational performance. Among them, resource integration capability has a mediation effect between organizational learning and organizational performance, it accounted for 0.166. Resource reconfiguration capability has a mediation effect between organizational learning and organizational performance, it accounted for 0.216. This conclusion shows that organizational learning cannot directly improve the organizational performance of human resource service enterprises, and the impact of organizational learning on organizational performance needs to be realized through the mediation effect of dynamic capability. At the same time, this conclusion effectively answers the question of “why organizational learning does not always lead to organizational performance improvement, and what kind of organizational learning can lead to innovative performance?”. Only the organizational learning that is consistent with the human resource service enterprises strategy and can be transformed by the dynamic capability of the enterprise, it can improve organizational performance.

Thirdly, environmental dynamics and its dimensions have a significantly positive moderation effect between dynamic capability and organizational performance of human resource service enterprises, which is basically consistent with the conclusions of the existing literature (Protogerou et al., 2012; Permana and Ellitan, 2020; Yoshikuni, 2021). However, the more valuable finding of this study is that two dimensions of environmental dynamics (technology environment and market environment) have a positive moderation effect between dynamic capability and organizational performance of human resource service enterprises. Among them, the moderation effect of technology environment between resource integration capability and organizational performance is 0.104, the moderation effect of technology environment between resource reconfiguration capability and organizational performance is 0.090, the moderation effect of market environment between resource integration capability and organizational performance is 0.104, the moderation effect of market environment on resource reconfiguration capability and organizational performance is 0.096. This results shows that the stronger the enterprise’s capability to perceive the market environment, the more fully able to tap and utilize potential market opportunities, and further improve the promotion effect of enterprise resource integration and reconfiguration on enterprise organizational performance. At the same time, this research conclusion provides an empirical basis for the multi-dimensional research perspective of the relationship between dynamic capability and organizational performance of human resource service enterprises.

## Conclusion

Based on the perspective of organizational learning, this study uses hierarchical linear regression and structural equation models to explore the impact mechanism of organizational learning on dynamic capability and organizational performance through survey data of enterprises in the national human resource service industrial park in China, verify the mediation effect of dynamic capability and the moderation effect of environmental dynamics. The main conclusions are as follows: Firstly, human resource service outsourcing and online recruitment are the mainstream service items of human resource service enterprises, service items such as mid-high end talent search and flexible employment are relatively weak. Secondly, organizational learning plays an important role in the dynamic capability of human resource service enterprises, organizational learning has a significantly positive impact on resource integration capability and resource reconfiguration capability. Thirdly, resource integration capability and resource reconfiguration capability have a significantly positive impact on the organizational performance of human resource service enterprises, and resource reconfiguration capability has a stronger impact on organizational performance than resource integration capability. Fourthly, resource integration capability and resource reconfiguration capability, respectively, play a partial mediation effect between organizational learning and human resource service enterprises organizational performance. Fifthly, technology environment has positively moderation effect between resource integration capability and organizational performance of human resource service enterprises, technology environment has positively moderation effect between resource reconfiguration capability and organizational performance of human resource service enterprises; market environment has positively moderation effect between resource integration capability and organizational performance, market environment has positively moderation effect between resource reconfiguration capability and organizational performance.

### Theoretical Contribution

Firstly, from the perspective of organizational learning theory and dynamic capability theory, it explains the positive impact mechanism of organizational learning on the dynamic capability of human resource service enterprises. In recent years, many scholars have focused on the internal mechanism of organizational learning on business models, institutional environment, knowledge integration, lacking the perspective of dynamic capability, and empirical research on human resource service enterprises. Ye and Chen (2019) emphasized that the research on the internal mechanism of organizational learning and organizational performance should be strengthened. From the perspective of organizational learning theory and dynamic capability theory, this study continues to learn and innovate, and explores the impact mechanism of organizational learning on resource integration and resource reconfiguration in dynamic capability.

Secondly, the introduction of dynamic capability as an mediation variable, comprehensively considered the impact of resource integration capability and resource reconfiguration capability in dynamic capability on organizational learning and organizational performance. Previous studies only examined the impact of organizational learning methods on organizational performance, and lacked discussion on integrating it with dynamic capability and human resource service enterprises organizational performance. This study better highlights organizational learning as a key element for advancing dynamic capability, integrates internal and external resources of the enterprise through big data, improves and innovates service products, obtains sustainable competitive advantages, and enhances organizational performance.

Thirdly, it reveals the moderation mechanism of market environment and technology environment in environmental dynamics between dynamic capability and organizational performance. One is the role of market environment, the behavior orientation of dynamic capability acting on organizational performance is moderated by market globalization, changes in customer needs, and customer satisfaction. Enterprises need to identify emerging markets and improve their dynamic adaptability to the market through learning. The second is the role of technology environment, the behavior orientation of dynamic capability acting on organizational performance is moderated by technology development trends and technology life cycles. Enterprises need to effectively integrate and reconstruct resources, master new technologies, improve organizational performance, and maximize the value of corporate resources.

### Practical Implications

This study has some practical implications for human resource service enterprises and managers to improve organizational performance. Firstly, we must pay more attention to the opening and investment of the mid-high end market, develop professional talent services, provide products and projects with high technical content, high information integration, and high value-added, which will help expand the business of human resource service enterprises, improve service quality and competitiveness, it can also prevent enterprises from falling into the quagmire of price wars.

Secondly, managers should pay attention to creating a good organizational learning atmosphere, encouraging employees to analyze and discussing the organization’s service projects (online recruitment, human resource service outsourcing and so on) in a timely manner, such as organizing learning and developing various forms of online recruitment channels. In addition, traditional recruitment websites, social platforms and mobile apps can be effectively used to increase interaction with job seekers, achieve precise matching of recommended positions, and organize employees to learn the information technology required for resource integration, thereby improving the overall learning capability of the organization.

Thirdly, enterprises should pay attention to the improvement of dynamic capability. Human resource service enterprises often need to face various information, material resources, financial resources, human resources and other resources in the development of human resources. Only when they are good at overall utilization can they be fully used by the enterprise, resource integration capability and resource reconfiguration capability can be improved to better establish and update communication. Network and sharing mechanism form a good interactive relationship between customers and enterprises, between enterprises and enterprises, so as to improve the organization performance of enterprises.

Fourthly, we must learn to deal with environmental changes. Human resource service enterprises should be customer-oriented, pay more attention to customer experience, segment the market, formulate personalized and differentiated service strategies for the needs of different enterprises, groups, and talents at different levels. Only in this way can we cope with the changes in technology environment and market environment, so as to provide more targeted “de-homogeneous” service products, enhance the competitiveness of enterprises, and promote the improvement of enterprise organizational performance.

### Limitations and Future Research Directions

This study has few limitations, which need to be acknowledged. Firstly, this study takes human resource service enterprises as the research object, and only considers the differences of human resource service enterprises, so subsequent studies can expand the scope of enterprises and consider traditional manufacturing into the research scope. Secondly, although this study sets enterprise scale, enterprise nature, enterprise service items, and the existence of technology R&D department as control variables, there are many factors that affect the performance of human resource service enterprises in reality, such as human resource service enterprises’ talent factors, brand factors and so on, future research can further consider controlling these influencing factors.

## Data Availability Statement

The original contributions presented in the study are included in the article/supplementary material, further inquiries can be directed to the corresponding author.

## Ethics Statement

Ethical review and approval was not required for the study on human participants in accordance with the local legislation and institutional requirements. Written informed consent from the patients/participants was not required to participate in this study in accordance with the national legislation and the institutional requirements.

## Author Contributions

SC and JZ designed the research and methodology, compiled the literature, and put forward the policy recommendations. SC provided guidance throughout the entire research process. Both authors contributed to the article and approved the submitted version.

## Conflict of Interest

The authors declare that the research was conducted in the absence of any commercial or financial relationships that could be construed as a potential conflict of interest.

## Publisher’s Note

All claims expressed in this article are solely those of the authors and do not necessarily represent those of their affiliated organizations, or those of the publisher, the editors and the reviewers. Any product that may be evaluated in this article, or claim that may be made by its manufacturer, is not guaranteed or endorsed by the publisher.
